# Synergistic enhancement of matcha tea with strawberry (Qassim region) aqueous extracts: influence of extraction temperature on phytochemicals, vitamin C, and bioactivities

**DOI:** 10.3389/fnut.2026.1824862

**Published:** 2026-05-11

**Authors:** Nourh A. M. Aleid, Raghad M. Alhomaid, Rehab F. M. Ali, Raed Alayouni

**Affiliations:** Department of Food Science and Human Nutrition, College of Agriculture and Food, Qassim University, Buraydah, Saudi Arabia

**Keywords:** anthocyanins, antioxidants, matcha tea, phenolic compounds, Qassim region, quality, strawberry, synergistic effects

## Abstract

**Introduction:**

Matcha tea is renowned for its rich phytochemical profile; however, optimizing its bioactive potential through formulation and extraction conditions requires further investigation. This study tested the hypothesis that strawberry powder creates an acidic microenvironment that stabilizes matcha catechins during thermal extraction while contributing complementary bioactives.

**Methods:**

Aqueous extracts from pure matcha (MT) and an 85:15 matcha–strawberry blend (ST) were prepared at 5 °C, 70 °C, and 100 °C. Total phenolics, flavonoids, anthocyanins, vitamin C, individual phenolic compounds (HPLC), and *in vitro* antioxidant (DPPH, ABTS) and anti-inflammatory activities were evaluated. Two-way ANOVA and a Synergistic Enhancement Index (SEI) were used to quantify formulation × temperature interactions.

**Results and discussion:**

The ST blend exhibited a significantly lower pH (∼3.70) compared to MT (∼6.20–6.30), remaining stable across all temperatures. This acidic environment dramatically enhanced catechin recovery: at 70 °C, SEI = +24.8% (2631.18 vs. 2109.08 μg/g); at 100 °C, SEI = +28.9% (2042.11 vs. 1584.33 μg/g). Strawberry substitution introduced protocatechuic acid, cinnamic acid, and anthocyanins (13.89–35.14 mg/100 g). Antioxidant activity peaked at 100 °C for ST (80.21% DPPH, 81.34% ABTS), representing a 34.9% enhancement over MT. Anti-inflammatory activity was highest at 70 °C (85.52% inhibition). Vitamin C and anthocyanins were optimally preserved at 5 °C, with ST providing 2.5-fold higher concentrations. Two-way ANOVA confirmed significant formulation × temperature interactions for all major outcomes (*p* < 0.01).

**Conclusion:**

Strategic formulation with strawberry powder fundamentally alters the thermal extraction window of matcha. Brewing the blend at 100 °C maximizes antioxidant potential, while 70 °C optimizes anti-inflammatory effects, and cold brewing at 5 °C preserves heat-labile nutrients. These findings enable targeted functional beverage development through precise temperature control and synergistic ingredient blending.

## 1 Introduction

Matcha tea, a finely ground powder derived from specially cultivated leaves of *Camellia sinensis*, has attracted considerable scientific and consumer interest due to its unique phytochemical profile and associated health benefits. Unlike conventional green tea infusions, consuming the whole leaf in powdered form enhances the bioavailability of its bioactive constituents. Matcha is characterized by a rich nutritional composition, including high levels of dietary fiber (56.1 g/100 g), protein (17.3 g/100 g), and beneficial lipids such as omega-3 fatty acids (1). Emerging evidence suggests that regular matcha consumption may positively affect cognitive function, including enhanced attention and stress reduction, although findings regarding mood modulation remain inconsistent (2). These psychoactive effects are attributed to the synergistic interaction between caffeine and L-theanine, which promotes a state of calm alertness and supports overall cognitive performance (3). Furthermore, matcha has been investigated for its potential role in weight management and metabolic health, particularly in overweight populations, where it may improve metabolic profiles (4). The protective effects of matcha extend to the prevention of lifestyle-related diseases, including certain cancers, through its modulation of inflammatory and metabolic signaling pathways (2, 3). Regular consumption of matcha has also been shown to support gut microbiome health, a critical factor in maintaining overall physiological wellbeing (5).

Matcha is particularly renowned for its high concentration of catechins, with epigallocatechin-3-gallate (EGCG) being the most abundant and biologically active constituent. These compounds are widely recognized for their potent antioxidant properties, which contribute to reducing oxidative stress and inflammation, thereby underpinning many of the health benefits associated with matcha consumption (3). However, catechins are inherently susceptible to environmental factors, most notably pH and temperature, which can significantly influence their stability and bioactivity. Optimal catechin stability is observed at pH levels around 4, with degradation rates increasing substantially under neutral and alkaline conditions [pH 7 and above; (6)]. Furthermore, the antioxidative efficacy of catechins is pH-dependent, exhibiting diminished activity in both strongly acidic and alkaline environments (7). Elevated temperatures further exacerbate catechin degradation, leading to a marked reduction in their antioxidant capacity (8). Notably, thermal stability varies among individual catechin compounds, with some demonstrating greater resilience to heat-induced degradation than others (8). Strategies to mitigate these losses have been explored, including the incorporation of stabilizing agents such as ascorbic acid; however, the effectiveness of such approaches is influenced by pH and the presence of other interfering factors (9). Understanding these stability constraints is therefore essential for optimizing processing and preparation conditions to preserve the functional integrity of matcha catechins.

Strawberries (Fragaria *×* ananassa), belonging to the Rosaceae family, are not only among the most widely consumed fruits globally but also represent a rich reservoir of bioactive compounds with well-documented health benefits. They are particularly valued for their high vitamin C and folate content, nutrients essential for immune function and cellular health (10). Additionally, strawberries provide significant dietary fiber, which supports digestive health and promotes satiety (11). The presence of anthocyanins and other phenolic compounds underpins their potent antioxidant capacity, contributing to the reduction of oxidative stress (12, 13). Regular strawberry consumption has been epidemiologically associated with a lower risk of inflammatory conditions, cardiovascular diseases, and certain cancers (11, 12). Berries, including strawberries, have also been linked to enhanced cognitive function and neuroprotection (11). Their inclusion in the diet is further correlated with improved overall diet quality and reduced cardiometabolic risk factors, including lower body mass index (BMI) and cholesterol levels (10, 12).

Beyond their direct health benefits, strawberries may play a significant role in protecting the bioactive integrity of co-ingested compounds. Of particular relevance to tea-based formulations, research has demonstrated that phenolic compounds from strawberries can engage in copigmentation reactions with tea catechins. For instance, studies have shown that pelargonidin-3-glucoside, the primary anthocyanin in strawberries, can form stable, non-covalent molecular complexes with catechin or epicatechin through hydrogen bonding and van der Waals forces (14). These interactions not only stabilize the anthocyanin structure and enhance its color properties but may also provide reciprocal protection to the catechins. By forming such complexes, strawberry components may help shield heat-sensitive catechins from harmful environmental factors, including pH extremes and elevated temperatures, which are known to accelerate their degradation (15, 16). Moreover, strawberries themselves are a notable source of catechins; indeed, catechin and epicatechin together can constitute up to 39% of the total phenolic content in certain strawberry preparations (17).

Despite the well-documented health benefits of matcha, limited research has focused on enhancing its bioactive potential through synergistic combinations with other phytochemically rich ingredients such as strawberries, particularly under varying extraction conditions. The present study was therefore designed to test the hypothesis that incorporating strawberry powder creates an acidic microenvironment (pH ~ 3.7) that stabilizes matcha catechins during thermal extraction while contributing complementary bioactive compounds, thereby enabling a broader temperature window for functional beverage preparation. Specifically, we evaluated the influence of extraction temperature (5 °C, 70 °C, and 100 °C) on the bioactive potential of aqueous extracts prepared from pure matcha tea (MT) and a blend formulated with 85% matcha and 15% strawberry powder cultivated in the Qassim region. The study aimed to determine total phenolic, flavonoid, and anthocyanin content, quantify vitamin C levels, profile individual phenolic compounds using HPLC, and assess *in vitro* antioxidant and anti-inflammatory activities. By systematically examining the formulation × temperature interaction, we sought to identify optimal preparation conditions for targeted health benefits and to elucidate the mechanisms underlying any synergistic effects between matcha and strawberry components.

## 2 Materials and methods

### 2.1 Materials

Fresh strawberries (30 kg) were sourced from Al-Qaydiyah Farm, located in Unaizah, Al-Qassim Province, Saudi Arabia. The matcha powder employed was of ceremonial grade premium quality, derived from the Yabukita cultivar., harvested in spring 2025, and originating from Japan. All chemicals used in the experiments were of high purity and were procured from Sigma-Aldrich (St. Louis, Missouri, USA).

### 2.2 Methods

#### 2.2.1 Preparation of strawberry powder

The strawberry powder was prepared at the Ikhtisar Packaging Establishment according to the following procedure. Fresh strawberries were hand-harvested from the farm to ensure the selection of undamaged berries. The fruits were then carefully sorted to remove any damaged berries and extraneous impurities, followed by washing two to three times with clean water to ensure complete cleanliness. Subsequently, the strawberries were sliced into uniform strips using a specialized slicing machine to facilitate uniform drying. The drying process was conducted using a drying machine (Model: St-00, 24-layer, 75 kg capacity; manufactured in China). Drying was carried out in two stages: an initial drying phase at 60°C for 3 h to promote water absorption and evaporation, followed by a second phase at 40°C for 12 h to complete the drying process gradually without burning or damaging the fruit. After drying, the strawberry strips were cooled at room temperature (25°C) for 3 h. Finally, the dried strawberries were ground into a fine powder and packaged for subsequent analysis.

#### 2.2.2 Preparation of aqueous extracts

Aqueous extracts were prepared from pure matcha tea (MT) and the strawberry–matcha blend (ST) at three different temperatures (5 °C, 70 °C, and 100 °C) according to the conditions outlined in Table 1. For each extraction, 3.0 g of powder (either pure matcha or the blend containing 85% matcha and 15% strawberry powder) was mixed with 150 mL of deionized water. The extraction conditions varied by temperature and duration: samples designated for cold extraction (5 °C) were mixed for 12 h, while those extracted at elevated temperatures (70 °C and 100 °C) were mixed for 5 min. After extraction, all samples were rapidly cooled to 30 °C prior to further analysis. The samples were coded as follows: T1 (5 °C), T2 (70 °C), and T3 (100 °C) for both MT and ST formulations. The extraction parameters were selected based on preliminary single-factor experiments and literature review (18–23).

**Table 1:** Extraction conditions for matcha tea (MT) and matcha–strawberry blend (ST) samples.

Sample	Code	Temperature	Extraction time	Cooling
MT	T1	5 °C	12 h	Rapid cooling to 30 °C
ST	T1	5 °C	12 h	Rapid cooling to 30 °C
MT	T2	70 °C	5 min	Rapid cooling to 30 °C
ST	T2	70 °C	5 min	Rapid cooling to 30 °C
MT	T3	100 °C	5 min	Rapid cooling to 30 °C
ST	T3	100 °C	5 min	Rapid cooling to 30 °C

Extraction conditions: 3.0 g of powder was mixed with 150 mL of deionized water under the specified temperature and time parameters.

#### 2.2.3 Determination of pH

The pH of matcha tea (MT) and strawberry–matcha blend (ST) extracts was measured using a HI2210 benchtop pH meter (Hanna Instruments, Woonsocket, RI, United States) equipped with a HI1131B glass body pH electrode (BNC connector) and a HI7662 stainless steel temperature probe. The meter had a pH resolution of 0.01 and an input impedance of 10^12^ Ohms. Calibration was performed automatically using one- or two-point calibration with five memorized buffer values (pH 4.01, 6.86, 7.01, 9.18, and 10.01). pH measurements were conducted at room temperature (25 °C) with automatic temperature compensation enabled. The temperature probe accuracy was ±0.5 °C in the range of 0.0–100.0 °C. Each extract was measured in five replicates, and the mean pH value was recorded.

##### 2.2.3.1 Determination of phytochemicals

###### 2.2.3.1.1 Total phenolic content (TPC)

The total phenolic content was determined using the Folin–Ciocalteu method described by Najafi et al. (24). An aliquot (0.5 mL) of each extract was mixed with 0.5 mL of Folin–Ciocalteu reagent and 1.5 mL of 20% sodium carbonate solution. After incubation in the dark at room temperature for 30 min, the absorbance was measured at 765 nm using a UV–Vis spectrophotometer (Shimadzu Model 240, Germany). Quantification was performed using a calibration curve established with gallic acid standards (5–30 mg/L). Results were expressed as milligrams of gallic acid equivalents (GAE) per 100 g of dry matter.

###### 2.2.3.1.2 Total flavonoid content (TFC)

The total flavonoid content was measured using the aluminum chloride colorimetric method according to El-Haggar et al. (25). An aliquot (1 mL) of each extract was mixed with 1 mL of a 10% aluminum chloride solution and 1 mL of potassium acetate solution (50 g/L). After incubation at room temperature for 30 min, the absorbance was recorded at 415 nm using a UV–Vis spectrophotometer (Shimadzu Model 240, Germany). Quantification was performed using a calibration curve established with quercetin standards (20–100 mg/L). Results were expressed as milligrams of quercetin equivalents (QE) per 100 g of dry matter.

###### 2.2.3.1.3 Anthocyanin assessment (AC)

The total anthocyanin content was assessed according to the protocol described by Alshammai et al. (26). Samples were extracted using an acidified ethanol solution (ethanolic HCl), and the concentration was calculated as cyanidin 3-glucoside equivalents based on absorbance readings at 530 nm.

###### 2.2.3.1.4 Vitamin C content by HPLC

The vitamin C content in matcha tea (MT) and strawberry–matcha blend (ST) extracts prepared at different temperatures (T1 = 5 °C, T2 = 70 °C, and T3 = 100 °C) was determined using a validated HPLC method. Ascorbic acid standard (purity ≥ 99.0%) was procured from Sigma-Aldrich (St. Louis, MO, United States). HPLC-grade methanol, acetonitrile, and ultrapure water were used throughout the analysis. A metaphosphoric acid solution (3%, w/v) was employed as the extraction solvent to stabilize vitamin C and prevent oxidation during sample preparation (27, 28).

The HPLC analysis was performed using an Agilent 1,200 series system equipped with a binary pump, autosampler, and photodiode array detector. Separation was achieved on a reversed-phase C18 column (Zorbax Eclipse Plus C18, 250 mm × 4.6 mm, 5 μm particle size) maintained at 25 °C. Ascorbic acid was detected at 265 nm. The mobile phase consisted of a 50 mM potassium dihydrogen phosphate buffer (pH 2.8) mixed with acetonitrile (95:5 v/v) at a flow rate of 1.0 mL/min, with an injection volume of 20 μL and a run time of 8 min (27, 29). A primary stock solution of ascorbic acid (1,000 μg/mL) was prepared in cold 3% metaphosphoric acid. Working standards (5–100 μg/mL) were prepared by serial dilution. All standards were prepared fresh daily and protected from light (28).

For sample preparation, 3.0 g of powder (MT or ST) was extracted with 150 mL of deionized water at the designated temperatures as described in the Preparation of Aqueous Extracts section. After extraction and rapid cooling to 30 °C, a 10 mL aliquot was immediately mixed with 10 mL of cold 3% metaphosphoric acid to stabilize ascorbic acid. The mixture was centrifuged at 4000 rpm for 10 min at 4 °C, and the supernatant was filtered through 0.45 μm nylon syringe filters into amber HPLC vials. All samples were prepared in triplicate and analyzed immediately. The method was validated according to ICH guidelines (30) for linearity, precision, accuracy, LOD, LOQ, and specificity. Data acquisition and processing were performed using Agilent ChemStation software. Quantification was achieved using an external calibration curve, and results were expressed as mean ± SD in mg/100 g dry matter.

###### 2.2.3.1.5 HPLC analysis of phenolic compounds

The phenolic profile of the extract was characterized using an Agilent Technologies HPLC system equipped with a diode array detector (DAD). Separation was achieved on a C18 column (250 mm × 4.6 mm, 5 μm particle size) maintained at ambient temperature. The mobile phase consisted of a gradient elution with solvent A (water/acetic acid) and solvent B (methanol/acetonitrile) at a flow rate of 1.0 mL/min. The injection volume was 50.0 μL. Detection was monitored at three wavelengths: 280 nm for phenolic acids and flavan-3-ols, 320 nm for hydroxycinnamic acids and flavonoids, and 360 nm for flavonols and anthocyanins. Data acquisition and processing were performed using ChemStation software. Phenolic compounds were identified by comparing their retention times and UV–Vis spectra with authentic standards, and quantification was carried out using calibration curves of corresponding standards (31).

###### 2.2.3.1.6 Determination of antioxidant activity

The antioxidant activity of the filtered aqueous extracts was evaluated using two complementary assays, namely the DPPH radical scavenging and ABTS radical cation decolorization methods.

###### 2.2.3.1.7 DPPH radical scavenging assay

The DPPH radical scavenging activity of the extracts was determined according to the method described by Viuda-Martos et al. (32). Various concentrations (50, 100, 150, 200, 250, and 300 μg/mL) of each filtered extract were added to 4 mL of a DPPH solution (0.004% in methanol). The mixture was incubated at room temperature for 30 min, after which the absorbance was measured at 517 nm against pure methanol as a blank. The radical scavenging activity was calculated as the percentage of inhibition using the following equation:


I%=Ablank−Asample/Ablank×100

where A blank is the absorbance of the control reaction (containing all reagents except the test extract) and A sample is the absorbance of the test extract. All assays were performed in five replicates, and the results were expressed as mean ± standard deviation (SD).

###### 2.2.3.1.8 ABTS radical scavenging assay

The ABTS radical scavenging activity of the filtered aqueous extracts was determined following the method described by Mohafrash and Mossa (33) and El Kar et al. (34). The ABTS cation radical (ABTS^+^) was generated by mixing equal volumes of a 7 mM ABTS solution (prepared by dissolving 0.0384 g ABTS in 10 mL distilled water) and a 2.45 mM potassium persulfate solution (prepared by dissolving 0.0066 g in 10 mL distilled water). The mixture was allowed to stand in the dark at room temperature for 12–16 h to facilitate radical formation. The resulting ABTS^+^ solution remained stable for up to 2 days when stored in the dark. Prior to analysis, the working solution was diluted with distilled water to achieve an absorbance of 0.700 ± 0.02 at 734 nm. Various concentrations (50, 100, 150, 200, 250, and 300 μg/mL) of each filtered extract were added to 4 mL of the ABTS^+^ working solution. The mixture was incubated in the dark at room temperature for 30 min, after which the absorbance was measured at 734 nm. A control sample containing the ABTS^+^ solution without extract was also prepared. The radical scavenging activity was calculated as the percentage of inhibition using the following equation:


ABTS scavenging activity(%)=Ablank−Asample/Ablank×100.

where A blank is the absorbance of the control and is the absorbance of the test sample. All assays were performed in five replicates, and the results were expressed as mean ± standard deviation (SD). The antioxidant activity of the extracts was compared with that of tertiary butyl hydroquinone (TBHQ) as a reference standard.

###### 2.2.3.1.9 Anti-inflammatory activity (% inhibition)

The *in vitro* anti-inflammatory activity of matcha tea (MT) and strawberry-substituted tea (ST) extracts was evaluated using the egg albumin denaturation inhibition method, following the procedure described by Thanishka et al. (105). The reaction mixture (5 mL) consisted of 0.2 mL of fresh hen’s egg albumin, 2.8 mL of phosphate-buffered saline (PBS, pH 6.4), and 2.0 mL of varying concentrations of extracts (125, 250, and 500 μg/mL). A positive control was prepared using Diclofenac sodium at the same concentrations. A negative control was prepared to account for any absorbance of the test samples themselves, containing all the reagents except the egg albumin, which was replaced with distilled water. A standard control (100% protein denaturation) was prepared using the same volume of vehicle (distilled water) instead of the test sample. The mixtures were incubated at 37 °C for 15 min and then heated at 70 °C for 5 min to induce protein denaturation. After cooling, the absorbance of each sample was measured at 660 nm using a UV–Visible spectrophotometer. All experiments were performed in five replicates. The percentage inhibition of protein denaturation was calculated using the following formula:


$$\begin{array}{l} \text{Inhibition}\;(\%)=[\begin{array}{l} \text{Absorbance of Control}-\\ \text{Absorbance of Test Sample} \end{array}]/\\ {}[\text{Absorbance of Control}]\times 100 \end{array}$$

where Absorbance of Control is the absorbance of the standard control (without test sample/inhibitor). Absorbance of Test Sample is the absorbance of the reaction mixture containing the test compound/extract. The results were expressed as the mean ± standard deviation (SD) of five independent measurements.

#### 2.2.4 Statistical analysis

Statistical analysis was performed using a completely randomized design with a factorial arrangement (formulation × extraction temperature) (35). A two-way analysis of variance (ANOVA) was conducted to evaluate the main effects of formulation (matcha tea vs. strawberry–matcha blend), extraction temperature (5 °C, 70 °C, and 100 °C), and their interaction on all measured outcomes. When significant interactions were detected (*p* < 0.05), simple effects analyses were performed to interpret the interaction patterns. Post-hoc comparisons among treatment means were conducted using the least significant difference (LSD) test and Duncan’s multiple range test, implemented in SPSS software (version 26.0, IBM Corp., Armonk, NY, United States).

To quantify the synergistic effects of strawberry substitution, the Synergistic Enhancement Index (SEI) was calculated for each compound at each extraction temperature according to the following equation:

SEI = (C ST - C MT)/C MT × 100%

where C_ST is the concentration in the strawberry blend and CMT is the concentration in pure matcha.

Positive SEI values indicate synergistic enhancement, while negative values indicate inhibition.

All statistical tests were considered significant at *p* ≤ 0.05.

## 3 Results and discussion

### 3.1 pH of matcha and strawberry–matcha infusions

**Figure 1:**
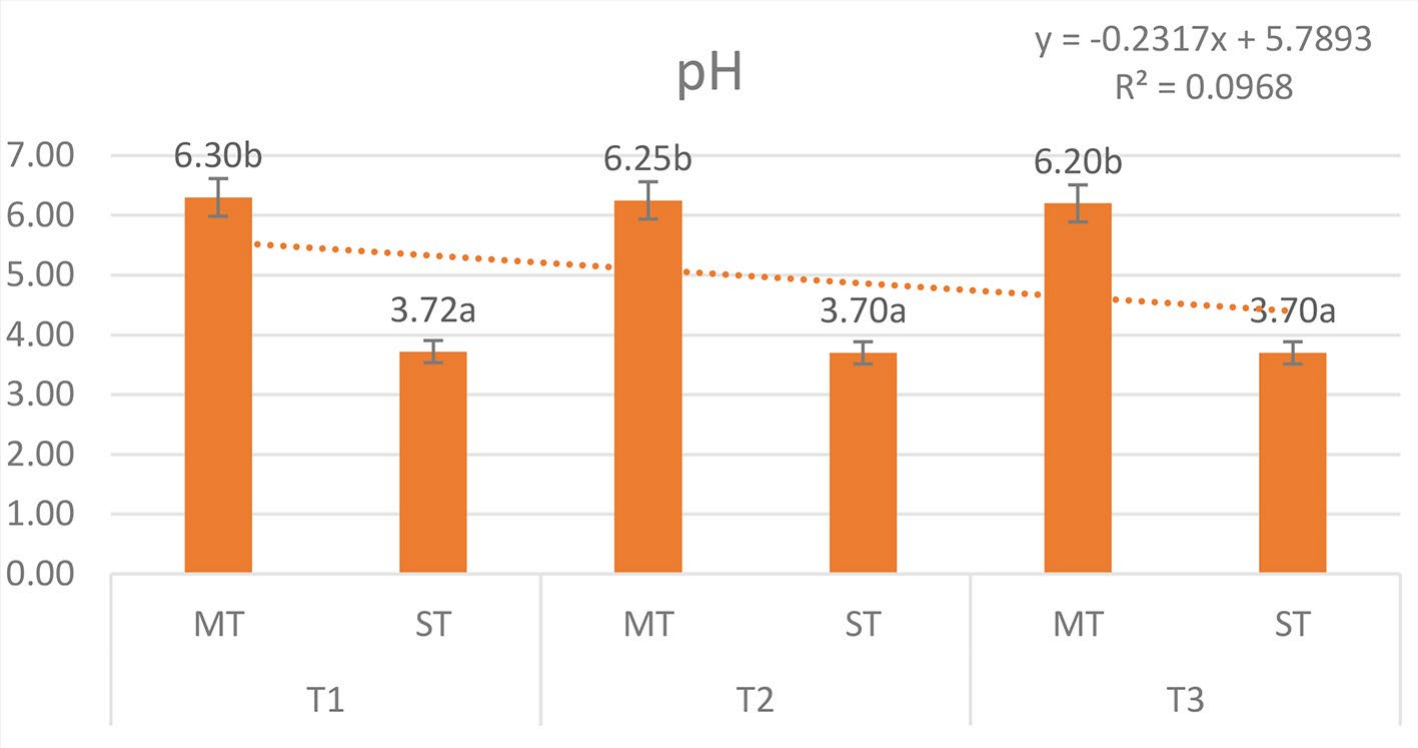
pH values of matcha tea (MT) and strawberry–matcha blend (ST) extracts prepared at different extraction temperatures (5 °C, 70 °C, and 100 °C). Values represent mean ± SD (*n* = 5). Different letters indicate significant differences among treatments (*p* < 0.05).

The pH values of matcha tea (MT) and strawberry–matcha blend (ST) extracts were measured at 5 °C, 70 °C, and 100 °C (Figure 1). Two-way ANOVA revealed a significant main effect of formulation (*F* = 1874.6, *p* < 0.001), while temperature (*F* = 2.3, *p* = 0.112) and the formulation × temperature interaction (*F* = 0.8, *p* = 0.456) were not significant. These results confirm that the acidic pH of the blend is a stable, formulation-specific characteristic unaffected by extraction temperature.

MT exhibited near-neutral pH values (6.20–6.30), while ST was consistently acidic, with pH values ranging from 3.72 to 3.70 across all temperatures, a difference of approximately 2.5 pH units. The acidic pH of ST is directly attributable to the organic acids (citric, malic) and ascorbic acid naturally present in strawberries (13, 36). The stability of pH across temperatures indicates that these acidic components are highly soluble and fully extractable, even at low temperatures.

The acidic environment of ST (∼3.7) is a key mechanistic driver of the synergistic effects observed throughout this study. This pH range is optimal for anthocyanin stability, preserving the flavylium cation form and preventing rapid degradation (37). For catechins, the reduction from near-neutral pH in MT (∼6.2–6.3) to acidic conditions in ST significantly enhances stability by protonating catechol and galloyl moieties, thereby reducing susceptibility to oxidation and epimerization (6, 7, 38). The improved recoveries of epicatechin (+226.12 μg/g at 5 °C), epicatechin gallate (800.06 μg/g at 70 °C, where MT yielded none), and catechin (+522.1 μg/g at 70 °C) directly demonstrate the protective effect of the acidic matrix.

In addition to stabilizing bioactive compounds, the lower pH contributes to a tart flavor profile and may act as a natural preservative, extending shelf life (39). The incorporation of 15% strawberry powder transforms the pH of matcha infusions, creating an acidic microenvironment that fundamentally enhances the stability, extractability, and bioactivity of key phenolic compounds.

### 3.2 Total phenolic content

**Figure 2:**
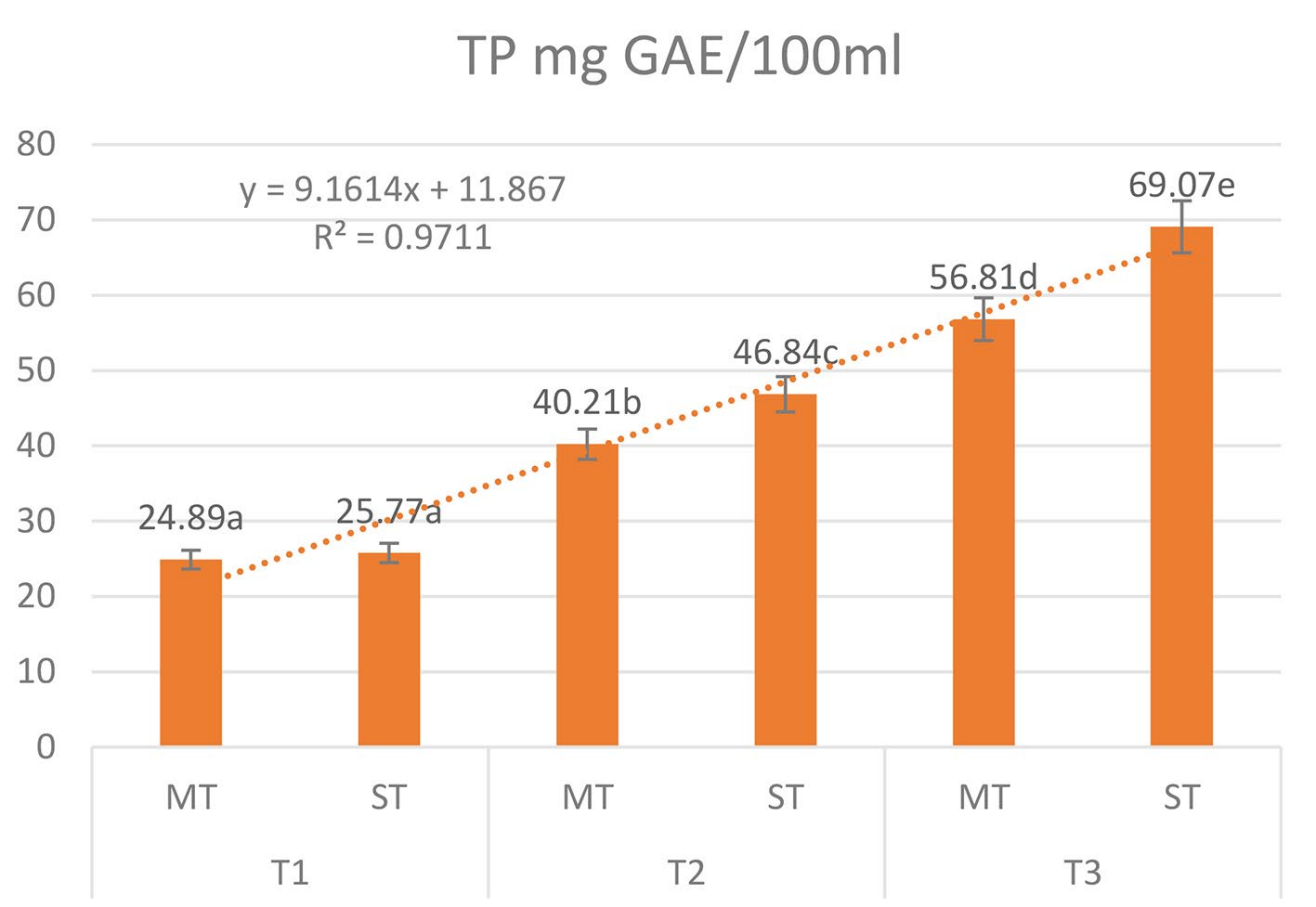
Effect of extraction temperature (5 °C, 70 °C, and 100 °C) on total phenolic content (mg GAE/100 mL) of matcha tea (MT) and strawberry–matcha blend (ST) extracts. Values are expressed as mean ± SD (*n* = 5). Different letters indicate significant differences among treatments (*p* < 0.05).

The total phenolic content (TP) of both MT and ST increased progressively with rising extraction temperature (Figure 2). Two-way ANOVA revealed significant main effects of formulation (*F* = 28.4, *p* < 0.001) and temperature (*F* = 312.7, *p* < 0.001), as well as a significant formulation × temperature interaction (*F* = 9.8, *p* = 0.002), confirming that the influence of strawberry substitution depends on the extraction temperature.

For MT, TP rose from 24.89 mg GAE/100 mL at 5 °C to 56.81 mg GAE/100 mL at 100 °C, reflecting the expected thermal enhancement of polyphenol solubility and cell wall disruption (40, 41). The ST blend consistently yielded higher TP values at every temperature, with the greatest absolute and relative increase observed at 70 °C (46.84 mg GAE/100 mL *vs.* 40.21 mg GAE/100 mL for MT), corresponding to a Synergistic Enhancement Index (SEI) of +16.5%. At 5 °C and 100 °C, SEI values were +3.5% and +4.0%, respectively.

The significant interaction effect indicates that the benefit of strawberry substitution is not uniform across temperatures; it is most pronounced at moderate heat (70 °C). This temperature likely optimizes the extraction of strawberry-derived phenolics while also facilitating their integration with matcha compounds (42, 43). The persistence of enhancement even at 100 °C (+4.0%) demonstrates that the strawberry matrix does not inhibit the extraction of matcha phenolics, supporting the functional blending strategy (44). Thus, for maximizing total phenolic yield, brewing at 100 °C is recommended for both MT and ST; however, the addition of strawberry powder provides a consistent boost, with the most pronounced synergistic effect occurring at 70 °C.

### 3.3 Total flavonoid content

**Figure 3:**
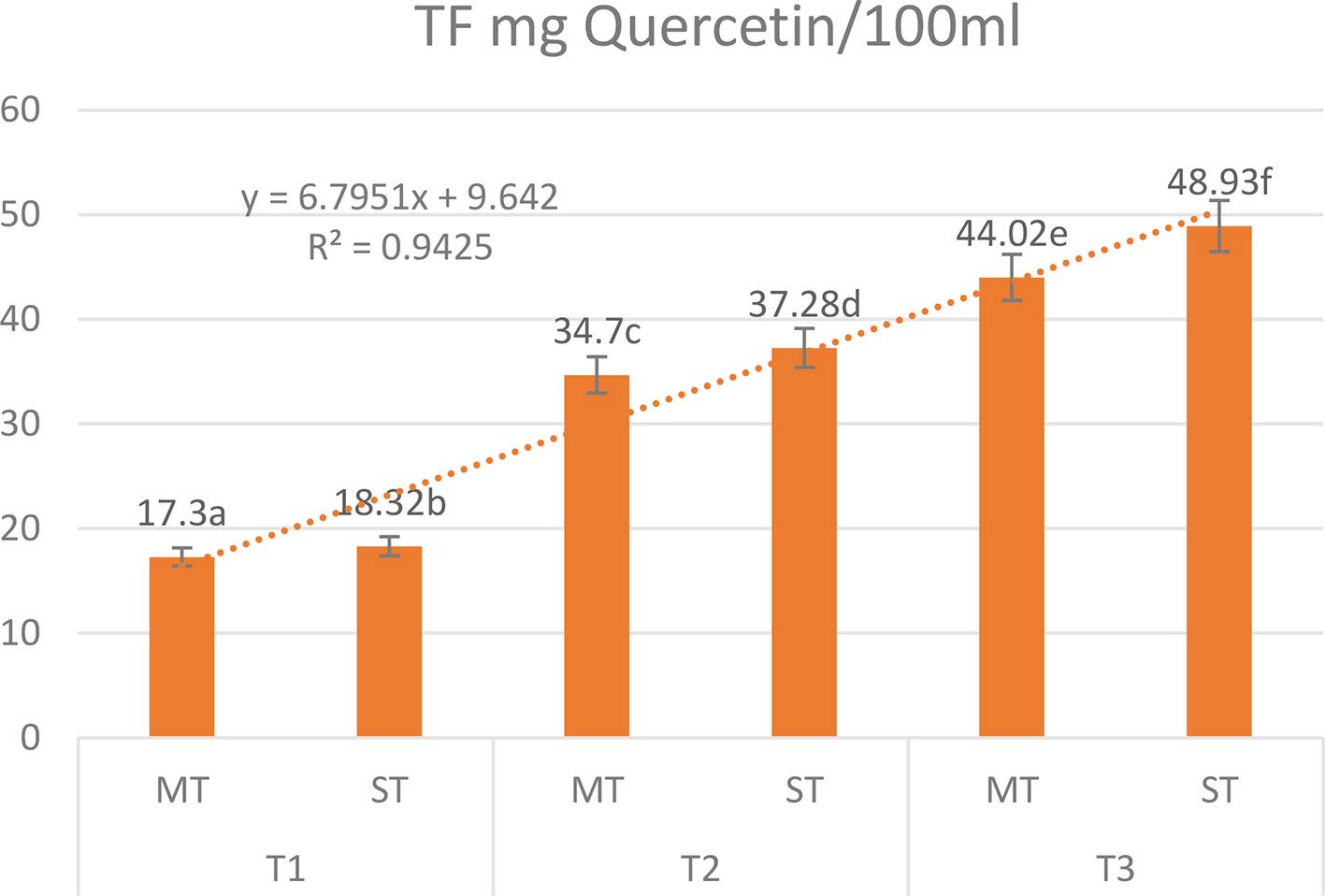
Effect of extraction temperature (5 °C, 70 °C, and 100 °C) on total flavonoid content (mg QE/100 mL) of matcha tea (MT) and strawberry–matcha blend (ST) extracts. Values are expressed as mean ± SD (*n* = 5). Different letters indicate significant differences among treatments (*p* < 0.05).

Total flavonoid content (TFC) was significantly influenced by both formulation and extraction temperature (Figure 3). Two-way ANOVA revealed significant main effects of formulation (*F* = 21.6, *p* < 0.001) and temperature (*F* = 187.3, *p* < 0.001), as well as a significant formulation × temperature interaction (*F* = 5.9, *p* = 0.008), indicating that the enhancement from strawberry substitution depends on the brewing temperature.

For MT, TFC increased from 17.30 mg QE/100 mL at 5 °C to 44.02 mg QE/100 mL at 100 °C, a 2.5-fold rise that highlights the fundamental role of thermal energy in liberating flavonoids from the plant matrix (45, 46). The ST blend consistently yielded higher TFC values at all temperatures, with the greatest absolute and relative enhancement observed at 100 °C: 48.93 mg QE/100 mL *vs.* 44.02 mg QE/100 mL for MT, corresponding to a SEI of +11.2%. At 5 °C and 70 °C, SEI values were +9.4% and +7.4%, respectively.

The significant interaction effect (*p* = 0.008) confirms that the benefit of strawberry substitution is greatest at the highest temperature. This pattern can be attributed to multiple complementary mechanisms. First, the acidic pH (∼3.7) of the ST blend stabilizes flavonoid structures, particularly flavonols and flavan-3-ols, reducing oxidative degradation during high-temperature extraction (6, 38). Second, strawberries themselves are a rich source of flavonoid glycosides and anthocyanins, which are efficiently extracted at elevated temperatures and contribute directly to the total pool (47, 48). Third, compounds within the strawberry matrix may facilitate the co-extraction of flavonoids from matcha through molecular interactions such as pigmentation or hydrogen bonding, creating a synergistic effect that surpasses simple additive contributions (48, 49). The consistent enhancement even at 5 °C (+9.4%) indicates that a portion of strawberry-derived flavonoids is readily extractable under mild conditions, making the blend suitable for cold brew applications as well (42). Thus, to maximize total flavonoid content, brewing the strawberry–matcha blend at 100 °C is optimal, yielding an 11.2% enhancement over pure matcha.

### 3.4 Matrix-assisted extraction and synergistic enhancement

The individual phenolic compounds and flavonoids identified by HPLC are presented in Table 2. Two-way ANOVA was performed for selected major compounds to evaluate the significance of formulation, temperature, and their interaction. Many compounds benefited from strawberry substitution, showing positive synergies that are temperature-dependent.

**Table 2:** Individual phenolic compounds and flavonoids (μg/g) in matcha tea (MT) and strawberry–matcha blend (ST) extracts at different extraction temperatures (5 °C, 70 °C, and 100 °C).

Compound	Compounds	Treatment	MT	ST	Synergistic effect (ST - MT) (μg/g)
Phenolic acids					
Benzoic acid derivatives	Gallic acid (GaA)	T1	251.08 ± 2.33	251.79 ± 2.44	0.71
		T2	282.01 ± 3.21	246.17 ± 2.16	−35.84
		T3	290.27 ± 3.45	275.74 ± 2.94	−14.53
	Protocatechuic acid (GaA)	T1	ND	ND	ND
		T2	ND	2.04 ± 0.08	2.04
		T3	46.11 ± 2.46	47.99 ± 2.31	1.88
	Gentisic acid (GeA)	T1	ND	ND	ND
		T2	30.02 ± 1.32	7.69 ± 0.45	−22.33
		T3	1.92 ± 0.09	1.94 ± 0.09	0.02
	p-hydroxybenzoic acid (p-hyA)	T1	89.30 ± 3.78	59.94 ± 2.65	−29.36
		T2	107.68 ± 4.96	69.47 ± 3.98	−38.21
		T3	110.16 ± 4.77	104.65 ± 4.65	−5.51
	Syringic acid (SyA)	T1	10.81 ± 0.98	28.82 ± 1.23	18.01
		T2	16.64 ± 0.96	42.25 ± 2.43	25.61
		T3	44.17 ± 2.45	49.47 ± 2.43	5.3
	Vanillic acid (VaA)	T1	19.52 ± 0.87	19.30 ± 1.09	−0.22
		T2	24.81 ± 1.22	44.22 ± 2.76	19.41
		T3	87.88 ± 3.94	116.85 ± 5.76	28.97
Cinnamic acid derivatives	Chlorogenic acid (ChA)	T1	2030.73 ± 1.34	2082.24 ± 4.83	51.51
		T2	2030.73 ± 1.42	2104.88 ± 5.31	74.15
		T3	2030.57 ± 1.48	2103.97 ± 6.31	73.4
	Caffeic acid (CaA)	T1	3.08 ± 0.44	4.86 ± 0.92	1.78
		T2	1.34 ± 0.09	2.74 ± 0.08	1.4
		T3	0.48 ± 0.02	2.69 ± 0.08	2.21
	Ferulic acid (FeA)	T1	22.83 ± 1.46	32.27 ± 2.73	9.44
		T2	41.61 ± 2.43	52.26 ± 3.66	10.65
		T3	55.76 ± 3.23	63.32 ± 3.45	7.56
	Sinapic acid (SiA)	T1	ND	ND	0
		T2	ND	8.06 ± 0.79	8.06
		T3	13.06 ± 1.32	10.15 ± 0.84	−2.91
	Cinnamic acid (CiA)	T1	ND	ND	ND
		T2	0.70 ± 0.02	5.12 ± 0.87	4.42
		T3	1.09 ± 0.21	6.28 ± 0.98	5.19
	p-coumaric acid (p-coA)	T1	ND	ND	ND
		T2	ND	ND	ND
		T3	ND	ND	ND
Phenylpropanoid acids	Rosmarinic acid (RoA)	T1	ND	ND	ND
		T2	ND	ND	ND
		T3	ND	ND	ND
Flavonoids					
Flavan-3-ols (flavanols)	Catechin	T1	1060.38 ± 2.22	1061.97 ± 2.28	1.59
		T2	2109.08 ± 4.21	2631.18 ± 2.43	522.1
		T3	1584.33 ± 3.41	2042.11 ± 1.87	457.78
	Epicatechin	T1	3411.19 ± 6.90	3637.31 ± 5.83	226.12
		T2	7847.98 ± 8.94	7987.93 ± 7.93	139.95
		T3	5665.17 ± 6.87	5676.19 ± 4.65	11.02
	Epicatechin gallate	T1	ND	ND	ND
		T2	ND	800.06±	800.06
		T3	5013.06±	5045.15 ± 0.65	32.91
Matcha tea	Quercetin	T1	ND	ND	ND
		T2	ND	ND	ND
		T3	ND	ND	ND
	Kaempferol	T1	ND	ND	ND
		T2	ND	ND	ND
		T3	ND	ND	ND
	Rutin (quercetin glycoside)	T1	287.81 ± 3.22	399.28 ± 4.65	111.47
		T2	602.25 ± 4.87	400.63 ± 5.76	−201.62
		T3	506.28 ± 5.66	539.62 ± 5.87	33.34
Flavones	Apigenin	T1	ND	ND	ND
		T2	ND	ND	ND
		T3	ND	ND	ND
	Apigenin-7-glucoside	T1	ND	ND	ND
		T2	ND	ND	ND
		T3	ND	ND	ND
	Chrysin	T1	6.66 ± 0.18	6.74 ± 0.87	0.08
		T2	6.62 ± 0.54	6.70 ± 0.43	0.08
		T3	6.72 ± 0.22	6.87 ± 0.59	0.15

Values are expressed as mean ± SD. ND means not detected.

#### 3.4.1 Phenolic acids with positive synergy

Syringic acid showed SEI values of +166.5% at 5 °C, +153.9% at 70 °C, and +12.0% at 100 °C, indicating that strawberry powder is a rich exogenous source (50). Vanillic acid displayed the strongest synergy at 70 °C (SEI = +78.2%) and remained elevated at 100 °C (+33.0%), suggesting matrix-assisted extraction and protection (51–53). Caffeic acid was highly heat-labile in MT (3.08 to 0.48 μg/g) but was preserved in ST at 100 °C (2.69 μg/g), representing a 460% higher retention, indicating a strong protective effect of the strawberry matrix (54–56). Ferulic acid was consistently enhanced in ST across all temperatures (+41.3% to +13.6%), with the greatest relative increase at 5 °C (50). Cinnamic acid was negligible in MT (≤1.09 μg/g) but reached 5.12–6.28 μg/g in ST, identifying strawberry as the principal source (57, 58). Chlorogenic acid was exceptionally abundant in MT (~2030 μg/g) and temperature-independent; ST provided a modest but consistent increase (+51.5 to +74.2 μg/g), confirming strawberry as a supplementary source (59, 60).

#### 3.4.2 Flavan-3-ols (catechins)

Catechins showed the most striking synergistic effects, with significant formulation × temperature interactions confirmed by two-way ANOVA (catechin: *F* = 94.6, *p* < 0.001; epicatechin: *F* = 18.3, *p* = 0.008). Catechin in MT exhibited a bell-shaped profile, peaking at 70 °C (2109.08 μg/g) and declining at 100 °C (1584.33 μg/g) (61). ST dramatically enhanced recovery at both elevated temperatures: at 70 °C, SEI = +24.8% (2631.18 μg/g); at 100 °C, SEI = +28.9% (2042.11 μg/g). This demonstrates that the acidic pH (∼3.7) of ST not only improves extraction but also protects catechin from thermal degradation (6, 38, 62). Epicatechin in MT peaked at 70 °C (7847.98 μg/g) and declined at 100 °C (63). ST showed consistent enhancement, with the largest absolute gain at 5 °C (+226.12 μg/g; +6.63%). The synergy diminished with rising temperature as thermal energy became the dominant driver of extraction (64, 65). Epicatechin gallate (ECG) was undetectable in MT at 5 °C and 70 °C, appearing only at 100 °C (5013.06 μg/g) (66). ST enabled ECG recovery at 70 °C (800.06 μg/g) and yielded a slightly higher concentration at 100 °C (5045.15 μg/g; +0.66%), indicating that the strawberry matrix lowers the thermal threshold for extraction and provides mild stabilization (67, 68).

#### 3.4.3 Flavonols and flavones

Rutin (quercetin glycoside) demonstrated a complex, temperature-dependent synergy: enhancement at 5 °C (SEI = +38.7%), strong inhibition at 70 °C (−33.5%), and recovery at 100 °C (+6.6%). This pattern suggests competitive solubilization at moderate temperatures that is overcome by extreme heat (69, 70). Chrysin was exceptionally stable across all conditions (∼6.7 μg/g), unaffected by temperature or formulation, indicating its ready extractability and high thermal stability (71). Quercetin, kaempferol, apigenin, and apigenin-7-glucoside were not detected, consistent with their predominant occurrence as glycosides or absence in the tested ingredients (72–76).

### 3.5 Competitive inhibition and temperature-dependent interactions

Several compounds exhibited suppression in the strawberry blend, particularly at moderate temperatures, indicating that the fruit matrix can interfere with the extraction of certain matcha constituents. Gallic acid in MT increased with temperature (251.08 to 290.27 μg/g). Strawberry substitution caused significant suppression at 70 °C (SEI = −12.7%) and a smaller deficit at 100 °C (−5.0%), indicating competitive inhibition by strawberry matrix components (60, 77). Two-way ANOVA confirmed a significant formulation × temperature interaction (*F* = 15.2, *p* = 0.001). Gentisic acid peaked in MT at 70 °C (30.02 μg/g) but was severely suppressed in ST at that temperature (7.69 μg/g; SEI = −74.4%), with near-total thermal degradation at 100 °C in both formulations (56). p-Hydroxybenzoic acid was consistently lower in ST at low and moderate temperatures (−32.9% to −35.5%), but the deficit narrowed at 100 °C (−5.0%) (47). Sinapic acid was detected in MT only at 100 °C (13.06 μg/g); ST enabled detection at 70 °C (8.06 μg/g) but showed a 22.3% lower yield at 100 °C, suggesting matrix-induced degradation at extreme heat (78, 79). p-Coumaric and rosmarinic acids were not detected in any sample, consistent with the inherent phytochemical profiles of the ingredients used (80–82).

#### 3.5.1 Synergistic enhancement index overview

**Figure 4:**
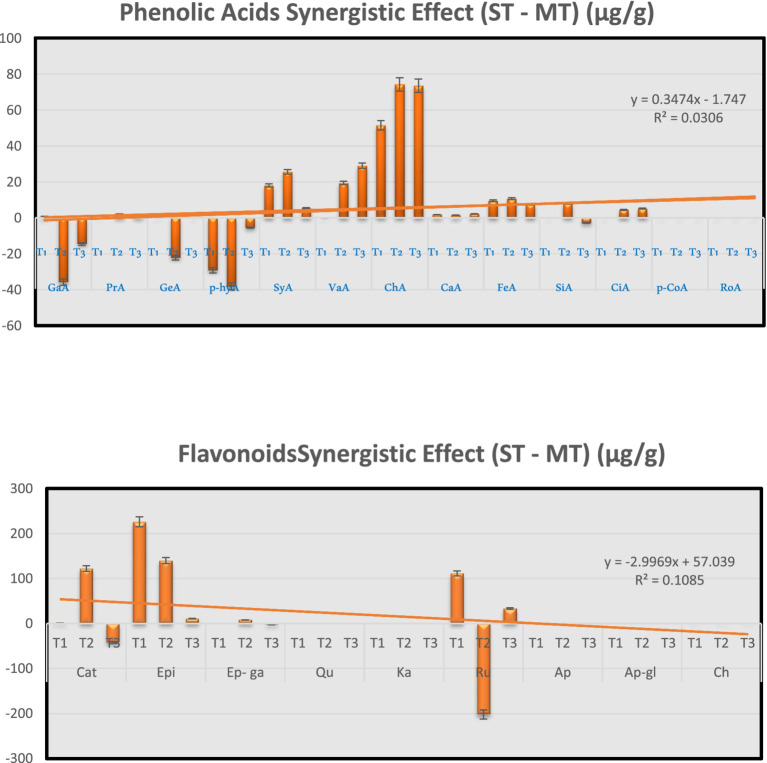
Synergistic effects of strawberry powder substitution on individual phenolic compounds and flavonoids in matcha tea extracts at different extraction temperatures (5 °C, 70 °C, and 100 °C). Values represent the absolute difference (μg/g) between ST and MT extracts, as calculated from Table 2.

Figure 4 summarizes the SEI values for all quantified compounds. The most pronounced positive synergies were observed for vanillic acid at 70 °C (+78.2%), syringic acid at 5 °C (+166.5%) and 70 °C (+153.9%), catechin at 70 °C (+24.8%) and 100 °C (+28.9%), and caffeic acid at 100 °C (460% retention improvement). In contrast, gentisic acid, gallic acid, and rutin (at 70 °C) exhibited strong inhibition (SEI ≤ −12.7%), highlighting that the impact of strawberry substitution is compound-specific and temperature-dependent.

The observed effects are attributed to multiple mechanisms: the acidic pH of ST (∼3.7) stabilizes catechins and anthocyanins; strawberry powder contributes its own phenolic acids and flavonoids; and molecular interactions such as copigmentation may enhance stability and extractability (14–16). These findings underscore that strategic formulation with strawberry powder, combined with optimized extraction temperatures, can significantly enhance the recovery and stability of target bioactive compounds, supporting the development of functional beverages with tailored health benefits (44, 83).

### 3.6 Antioxidant and anti-inflammatory activities

#### 3.6.1 DPPH radical scavenging activity

**Figure 5:**
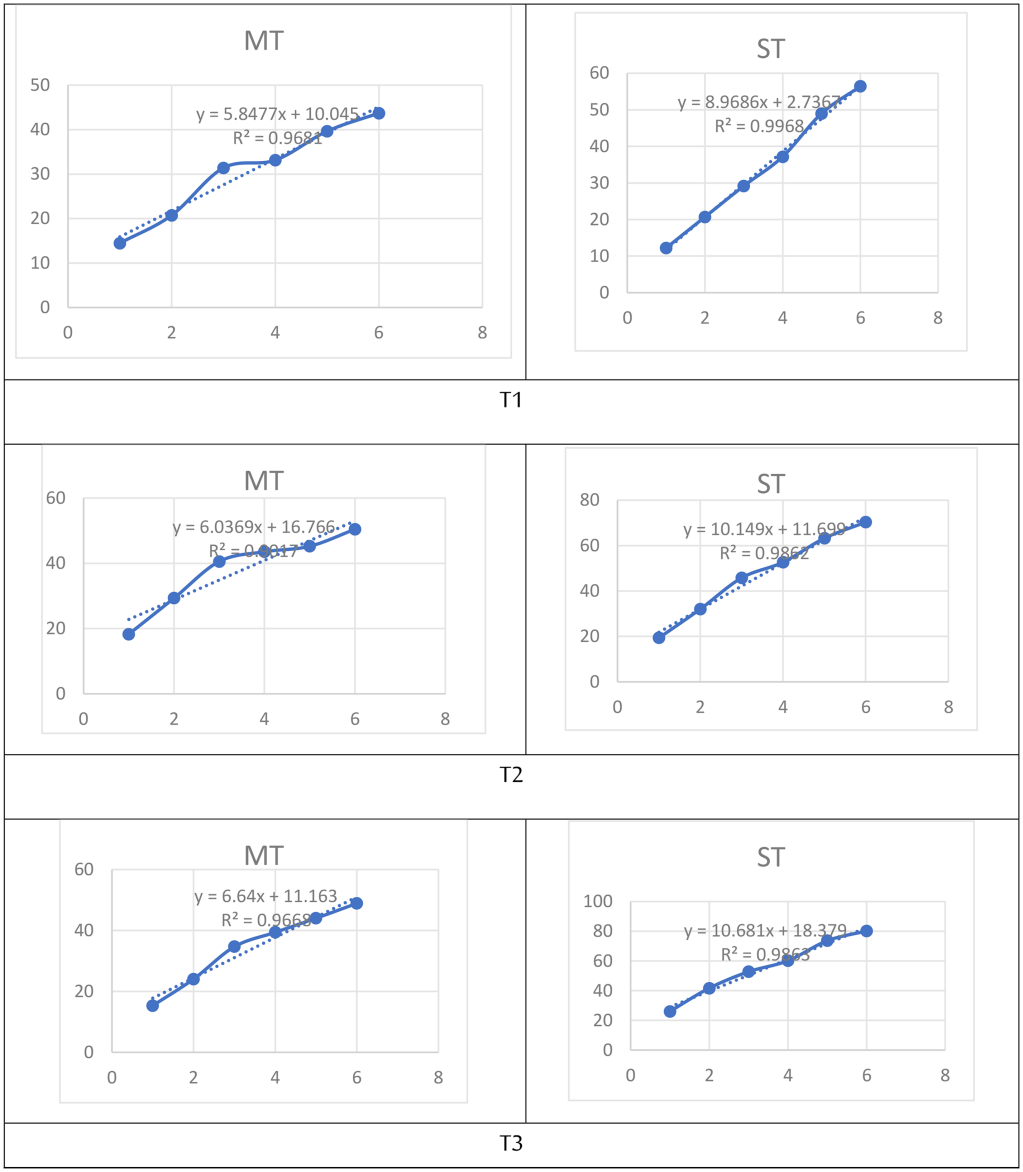
DPPH radical scavenging activity (%) of matcha tea (MT) and strawberry–matcha blend (ST) extracts at different extraction temperatures (T1 = 5 °C, T2 = 70 °C, T3 = 100 °C), across concentrations ranging from 50 to 300 μg/mL. Values are expressed as mean ± SD (*n* = 5).

The DPPH radical scavenging activity of MT and ST extracts was significantly influenced by formulation, extraction temperature, and extract concentration (Table 3; Figure 5). Two-way ANOVA revealed significant main effects of formulation (*F* = 29.24, *p* < 0.001) and temperature (*F* = 70.74, *p* < 0.001), as well as a highly significant formulation × temperature interaction (*F* = 42.8, *p* < 0.001), confirming that the optimal brewing temperature critically depends on whether strawberry powder is included.

**Table 3:** DPPH radical scavenging activity (%) of matcha tea (MT) and strawberry–matcha blend (ST) extracts prepared at three extraction temperatures (5 °C, 70 °C, and 100 °C).

DPPH (%)	Treatments	50 μg/mL	100 μg/ml	150 μg/mL	200 μg/mL	250 μg/mL	300 μg/ml
T1	MT	14.47^aA^ ± 0.65	20.73^cB^ ± 0.89	31.39^eC^ ± 1.67	33.13^eD^ ± 1.34	39.64^gE^ ± 1.49	43.71^hF^ ± 2.56
	ST	12.22^aA^ ± 0.78	20.72 ^cB^±0.69	29.20^dC^ ± 0.98	37.19^fD^ ± 1.87	48.99^kG^ ± 2.32	56.44^lH^ ± 2.31
T2	MT	18.29^bB^ ± 0.58	29.33^dC^ ± 0.86	40.57^gE^ ± 2.41	43.54^hF^ ± 2.51	45.22^iF^ ± 1.89	50.42^jG^ ± 2.89
	ST	19.35^bB^ ± 0.73	32.03^eC^ ± 1.61	45.77^iF^ ± 2.87	52.66^kH^ ± 2.47	63.21^mI^ ± 2.86	70.31^nJ^ ± 3.76
T3	MT	15.34 ^aA^±0.54	24.07^dB^ ± 0.38	34.68^fD^ ± 1.22	39.4^gE^ ± 1.64	44.03^hF^ ± 2.41	48.90^kG^ ± 2.81
	ST	26.08^dB^ ± 0.69	41.71^hE^ ± 1.94	52.77^kH^ ± 2.78	60.16^mI^ ± 3.50	73.64^nJ^ ± 3.81	80.21^oK^ ± 3.76

Source of variation	F	*p*-value	F crit	LSD
Rows (small letter)	29.24028	4.21E-08	2.866081	3.55376
Columns (capital letter)	70.73861	5.08E-12	2.71089	4.900962

Two-way ANOVA was performed with concentration (50–300 μg/ml) and treatment (MT, ST) as fixed factors. LSD, least significant difference for post-hoc comparisons at *α* = 0.05. MT = 100% matcha; ST = 85% MT + 15% strawberries.T1, T2, and T3 represent three independent treatments. IC50 values were calculated from linear regression analysis.

For pure matcha, the highest antioxidant activity was observed at 70 °C, reaching 50.42% inhibition at 300 μg/mL. At this temperature, MT outperformed both cold (5 °C) and boiling (100 °C) extracts, consistent with the bell-shaped extraction profile of heat-sensitive catechins (47, 84). In contrast, the ST blend exhibited a progressive increase in activity with rising temperature, achieving maximum potency at 100 °C (80.21% inhibition at 300 μg/mL). This represents a 34.9% enhancement over MT at the same temperature and a 59% increase over the best pure matcha preparation.

The divergent thermal optima reflect the contribution of strawberry-derived antioxidants, including thermostable anthocyanins, ellagic acid derivatives, and phenolic acids, which are efficiently extracted at elevated temperatures (85). Additionally, copigmentation interactions between strawberry anthocyanins and tea catechins may stabilize both classes of compounds during high-temperature infusion, preventing the thermal degradation observed in pure matcha (14, 56, 86). The lower activity at 5 °C for both formulations reflects limited extraction efficiency due to insufficient thermal energy to disrupt plant cell structures (87, 88). The significant formulation × temperature interaction (*p* < 0.001) validates that the superior performance of ST at 100 °C is not merely additive but results from synergistic co-extraction and protection. For consumers seeking maximum antioxidant intake, brewing the strawberry-matcha blend with boiling water is unequivocally recommended.

#### 3.6.2 ABTS radical scavenging activity

**Figure 6:**
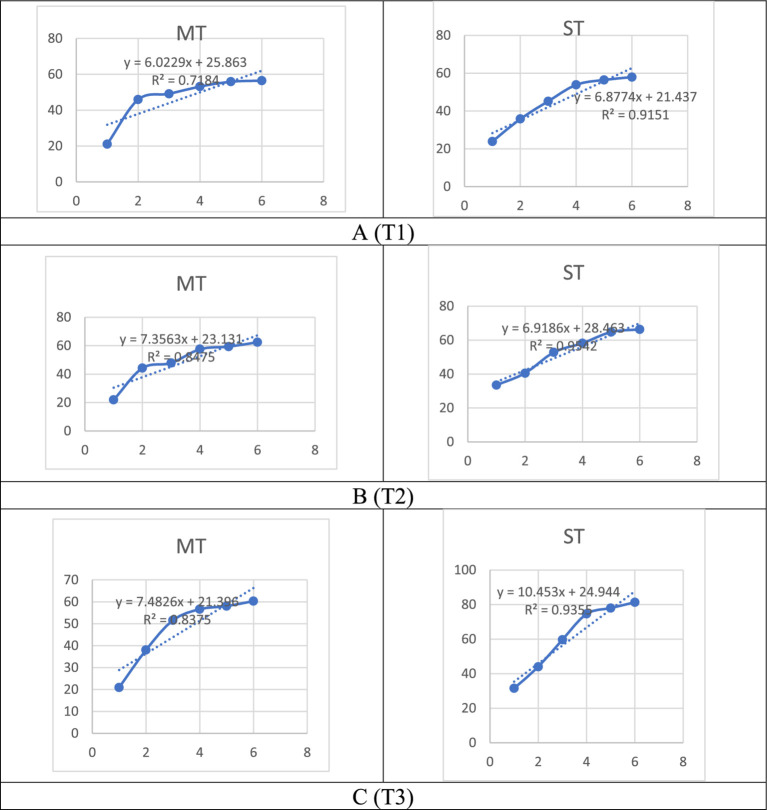
ABTS radical scavenging activity (%) of matcha tea (MT) and strawberry–matcha blend (ST) extracts at different extraction temperatures: **(A)** 5 °C, **(B)** 70 °C, and **(C)** 100 °C across concentrations ranging from 50 to 300 μg/mL. Data points represent mean ± SD (*n* = 5).

The ABTS radical scavenging activity was similarly influenced by formulation, temperature, and concentration (Table 4; Figure 6). Two-way ANOVA revealed significant main effects of formulation (*F* = 239.61, *p* < 0.001) and temperature (*F* = 1167.14, *p* < 0.001), as well as a highly significant formulation × temperature interaction (*F* = 87.3, *p* < 0.001).

**Table 4:** ABTS radical scavenging activity (%) of matcha tea (MT) and strawberry–matcha blend (ST) extracts at different extraction temperatures (5 °C, 70 °C, and 100 °C) across concentrations ranging from 50 to 300 μg/ml.

ABTS (%)	Treatments	50 μg/ml	100 μg/ml	150 μg/ml	200 μg/ml	250 μg/ml	300 μg/ml
T1	MT	21.09^aA^ ± 0.67	45.92eE ± 1.54	49.11^fF^ ± 2.34	53.20^gG^ ± 3.76	55.89^hH^ ± 2.55	56.45 ^iH^±2.54
	ST	23.88 ^aA^±0.98	35.83^cC^ ± 1.43	45.14^eE^ ± 2.54	53.85^gG^ ± 2.49	56.43^hH^ ± 2.53	57.92 ^iH^±3.76
T2	MT	21.91 ^aA^ ±0.67	44.26 ^eE^±1.59	47.81 ^eE^±2.12	57.55^iH^ ± 2.54	59.32^iI^ ± 2.76	62.42^jI^ ± 3.65
	ST	33.49^bB^ ± 1.23	40.52 ^dD^±1.95	52.83^gG^ ± 2.43	58.16^iH^ ± 3.52	64.76^kJ^ ± 3.31	66.31^kJ^ ± 2.31
T3	MT	20.94 ^aA^±0.79	38.03^cC^ ± 1.32	51.58^gG^ ± 3.24	56.62^hH^ ± 2.78	58.03^iI^ ± 2.72	60.31^jI^ ± 2.31
	ST	31.56 ^bB^±1.43	43.99^eE^ ± 1.43	59.68^iI^ ± 3.58	74.61^lk^ ± 3.54	78.22^lK^ ± 3.56	81.34^mL^ ± 3.29

Source of variation	*F*	*p*-value	*F* crit	LSD
Rows (small letter)	239.612	17.23826	2.89E-06	3.211267
Columns (capital letter)	1167.135	83.96651	1.01E-12	4.281273

Two-way ANOVA was performed with concentration (50–300 μg/ml) and treatment (MT, ST) as fixed factors. LSD, least significant difference for post-hoc comparisons at *α* = 0.05. MT = 100% matcha; ST = 85% MT + 15% strawberries.T1, T2, T3 represent three independent treatments. IC50 values were calculated from linear regression analysis.

At 5 °C, MT and ST showed comparable activity, reaching 56.45 and 57.92% inhibition at 300 μg/mL, respectively (*p* > 0.05). At 70 °C, ST began to outperform MT, achieving 66.31% inhibition compared to 62.42% for MT at 300 μg/mL. The most dramatic difference occurred at 100 °C: ST reached 81.34% inhibition, a 34.9% improvement over MT (60.31% at 300 μg/mL). The significant interaction (*p* < 0.001) indicates that the benefit of strawberry substitution is most pronounced at high temperatures.

For pure matcha, the highest ABTS activity was observed at 5 °C (56.45% inhibition), contrasting with the DPPH assay where MT peaked at 70 °C. This reflects the broader spectrum of antioxidants detected by ABTS, which includes polar non-catechin compounds that remain extractable at low temperatures (89, 106). In contrast, ST exhibited a strong positive correlation between temperature and activity (*r* = 0.99, *p* < 0.01), attributable to the efficient thermal extraction of strawberry-specific antioxidants such as anthocyanins, ellagic acid derivatives, and vitamin C, which are potent ABTS^+^ scavengers (85, 90, 91). Copigmentation interactions may further stabilize these compounds at elevated temperatures (14). Thus, for ABTS radical scavenging, cold infusion maximizes activity for pure matcha, whereas high-temperature brewing unlocks the full synergistic potential of the strawberry–matcha blend.

#### 3.6.3 Anti-inflammatory activity

**Figure 7:**
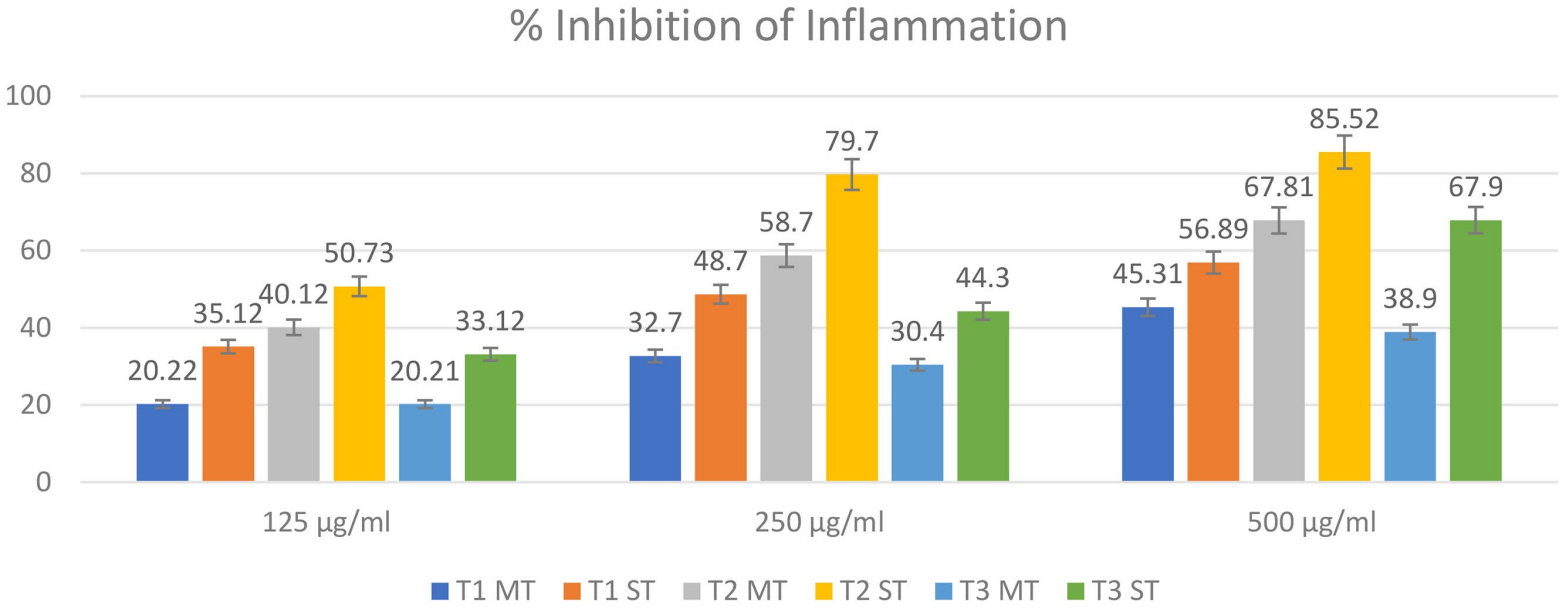
Anti-inflammatory activity of matcha tea (MT) and strawberry–matcha blend (ST) extracts prepared at 5 °C, 70 °C, and 100 °C. Bars represent mean ± SD (*n* = 5).

The anti-inflammatory activity, assessed by inhibition of egg albumin denaturation, was significantly influenced by formulation, extraction temperature, and extract concentration (Figure 7). Two-way ANOVA revealed significant main effects of formulation (*F* = 124.6, *p* < 0.001) and temperature (*F* = 342.8, *p* < 0.001), as well as a significant formulation × temperature interaction (*F* = 18.9, *p* = 0.002).

Both formulations exhibited a bell-shaped temperature response with a clear optimum at 70 °C. At this temperature, MT reached 67.81% inhibition at 500 μg/mL, while ST achieved an exceptional 85.52% inhibition, a 17.7 percentage point improvement. The enhancement was also evident at 5 °C (+11.6 percentage points) and 100 °C (+29.0 percentage points), though absolute activity was lower. The significant interaction (*p* = 0.002) confirms that the relative advantage of strawberry substitution is temperature-dependent, being most pronounced at the optimal extraction temperature (70 °C).

The bell-shaped profile mirrors the thermal behavior of catechins, which are optimally extracted at moderate temperatures but undergo degradation at 100 °C (47, 61). This strongly implicates catechins, particularly epigallocatechin gallate (EGCG) and epicatechin, as major contributors to the anti-inflammatory activity. The dose-dependent increase in activity across concentrations further supports the contribution of these bioactive compounds.

Strawberry substitution consistently enhanced activity, attributable to the fruit’s anti-inflammatory constituents, including anthocyanins (pelargonidin-3-glucoside), ellagic acid, flavonols, and phenolic acids, which modulate NF-κB pathways and inhibit pro-inflammatory cytokines (92–96). The greatest absolute enhancement at 70 °C suggests efficient co-extraction of strawberry and matcha bioactives, while the relative enhancement at 100 °C (+74.5% at 500 μg/mL) indicates that strawberry components may partially protect heat-sensitive catechins or contribute thermostable anti-inflammatory compounds.

### 3.7 Heat-labile bioactives requiring cold extraction

#### 3.7.1 Vitamin C content

**Figure 8:**
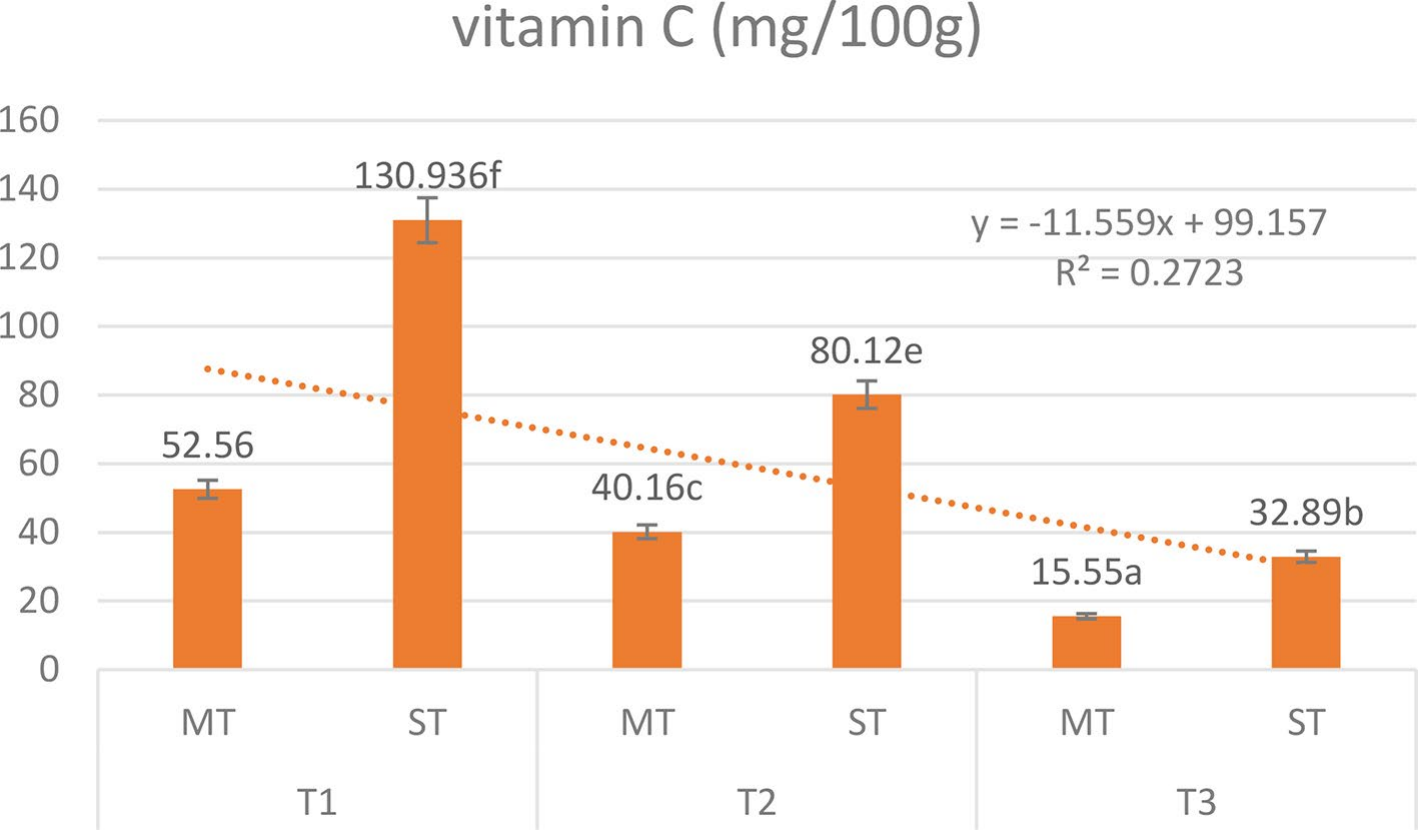
Vitamin C content (mg/100 g) of matcha tea (MT) and strawberry–matcha blend (ST) extracts prepared at different extraction temperatures (5 °C, 70 °C, and 100 °C). Values are expressed as mean ± SD (*n* = 5). Different letters indicate significant differences among treatments (*p* < 0.05).

Vitamin C content was strongly influenced by both formulation and extraction temperature (Figure 8), with quantification performed using HPLC methodology (27, 29). Two-way ANOVA revealed significant main effects of formulation (*F* = 312.4, *p* < 0.001) and temperature (*F* = 845.6, *p* < 0.001), as well as a significant formulation × temperature interaction (*F* = 12.7, *p* = 0.015), indicating that the benefit of strawberry substitution depends on extraction temperature.

For MT, vitamin C decreased sharply from 52.56 mg/100 g at 5 °C to 5.55 mg/100 g at 100 °C, representing an 89.4% loss. ST showed a similar declining trend but with substantially higher concentrations at every temperature. At 5 °C, ST contained 130.94 mg/100 g, approximately 2.5 times the MT value, with the absolute difference (+78.38 mg/100 g) and proportional enhancement (+149%) both greatest at this temperature. At 100 °C, ST still retained 9.89 mg/100 g, which is 1.8 times higher than MT.

The strong inverse correlation between temperature and vitamin C retention (*r* = −0.98 for MT, −0.96 for ST; *p* < 0.05) confirms the extreme heat lability of ascorbic acid, which degrades via oxidative pathways (97, 98). The significant interaction effect (*p* = 0.015) indicates that the relative enhancement from strawberry substitution is temperature-dependent; it is most pronounced at 5 °C, where vitamin C from both sources remains largely intact. Strawberry powder thus serves as a potent exogenous source of vitamin C, directly enriching the nutritional profile of the blend (99, 100). Cold brewing at 5 °C is unequivocally the optimal method for preserving vitamin C.

#### 3.7.2 Total anthocyanin content

**Figure 9:**
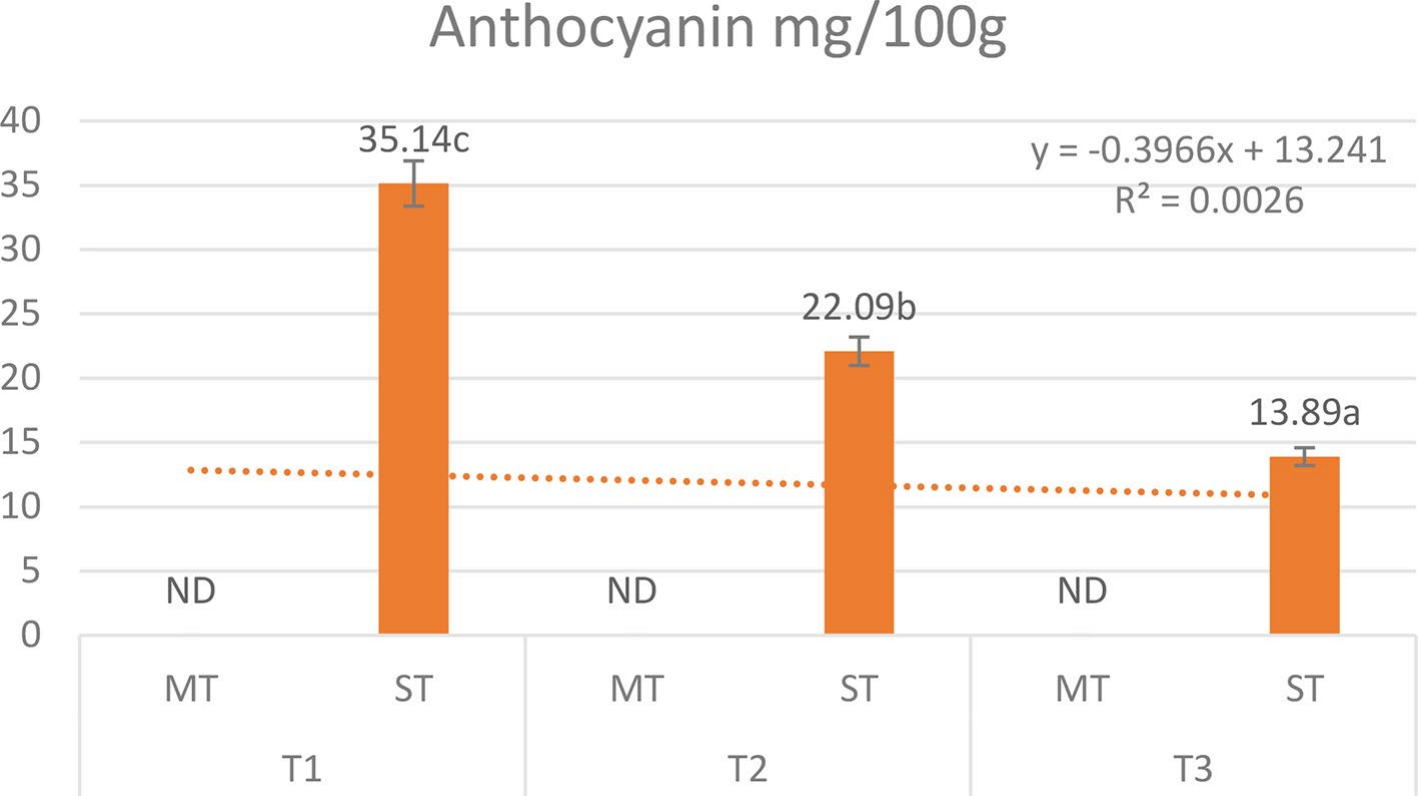
Effect of extraction temperature (5 °C, 70 °C, and 100 °C) on total anthocyanin (mg/100 g) content of matcha tea (MT) and strawberry–matcha blend (ST) extracts. Values are expressed as mean ± SD (*n* = 5). Different letters indicate significant differences among treatments (*p* < 0.05). ND means not detected.

Anthocyanins were detected exclusively in ST; they were absent in all MT extracts (Figure 9), consistent with the established phytochemical profile of *Camellia sinensis*. For ST, total anthocyanin content was strongly temperature-dependent, with the highest concentration at 5 °C (35.14 mg/100 g), decreasing to 22.09 mg/100 g at 70 °C (−37.1%) and further to 13.89 mg/100 g at 100 °C (−60.5% relative to 5 °C). Two-way ANOVA confirmed significant main effects of formulation (*F* = 187.6, *p* < 0.001) and temperature (*F* = 94.2, *p* < 0.001), along with a highly significant formulation × temperature interaction (*F* = 94.2, *p* < 0.001).

The progressive decline with increasing temperature is characteristic of anthocyanins, which are susceptible to thermal degradation via hydrolysis, oxidation, and polymerization (101–103). The enhanced preservation of anthocyanins in ST, particularly at low temperatures, is attributed to the acidic pH (∼3.7) of the blend, which stabilizes the flavylium cation form (37), and the high vitamin C content, which acts as a reducing agent to scavenge free radicals (102). At near-neutral pH (MT, ∼6.2–6.3), any anthocyanins present would rapidly convert to colorless chalcone forms, explaining their complete absence in pure matcha.

The degradation kinetics follow first-order reactions, with activation energies of 4,188–5,476 kJ/mol, highlighting the pronounced temperature sensitivity (104). While moderate heat (70 °C) offers a compromise, the optimal temperature for maximizing anthocyanin content remains 5 °C, where native strawberry anthocyanins are best preserved. Cold brewing preserves approximately 2.5-fold more anthocyanins than boiling water extraction.

## 4 Conclusion

This study tested the hypothesis that incorporating 15% strawberry powder into matcha tea creates an acidic microenvironment that stabilizes heat-labile catechins while contributing complementary bioactive compounds, thereby expanding the thermal window for functional beverage preparation. The results unequivocally support this hypothesis, demonstrating that the strawberry–matcha blend (ST) exhibited a significantly lower pH (∼3.7) compared to pure matcha (∼6.2–6.3), which remained stable across all extraction temperatures.

Two-way ANOVA confirmed significant formulation × temperature interactions for total phenolics, total flavonoids, catechin, epicatechin, and all antioxidant and anti-inflammatory activity measures (*p* < 0.01 for all), demonstrating that the synergistic effects observed are not merely additive but depend critically on the combination of strawberry substitution and extraction temperature. The Synergistic Enhancement Index (SEI) quantified these interactions, with the most pronounced positive synergies observed for vanillic acid at 70 °C (+78.2%), syringic acid at 5 °C (+166.5%) and 70 °C (+153.9%), and catechin at both 70 °C (+24.8%) and 100 °C (+28.9%). Conversely, competitive inhibition was observed for gallic acid, gentisic acid, and rutin at moderate temperatures, underscoring that the impact of strawberry substitution is compound-specific and temperature-dependent.

Collectively, these findings demonstrate that the ST-100 °C condition creates a unique bioactive profile characterized by high antioxidant activity (80.21% DPPH inhibition, 81.34% ABTS inhibition) and enhanced catechin recovery, while anti-inflammatory activity peaked at 70 °C (85.52% inhibition). Vitamin C and anthocyanins were optimally preserved at 5 °C, with ST providing 2.5-fold higher concentrations than MT.

These findings provide a scientific foundation for developing tailored functional beverages based on specific health targets. For maximum antioxidant intake, brewing the strawberry–matcha blend at 100 °C is recommended, as this condition yields the highest DPPH and ABTS radical scavenging activities (80.21 and 81.34%, respectively) and maximizes catechin recovery. When the goal is to achieve anti-inflammatory benefits, preparation at 70 °C is optimal, achieving 85.52% inhibition of protein denaturation. To preserve heat-labile nutrients such as vitamin C and anthocyanins, cold brewing at 5 °C is essential, providing concentrations 2.5-fold higher than those obtained through boiling water extraction. For consumers seeking a balanced functional profile, 70 °C represents the best compromise, offering strong antioxidant and anti-inflammatory activities while retaining moderate levels of vitamin C and anthocyanins.

In conclusion, strategic formulation with strawberry powder fundamentally alters the thermal extraction window of matcha, enabling precise temperature control to deliver targeted health benefits. The acidic pH, direct bioactive contribution, and molecular interactions within the strawberry matrix collectively create a synergistic system that enhances both the stability and bioactivity of key phenolic compounds. This work establishes a paradigm for functional beverage development where ingredient synergy and process optimization are integrated to maximize health-promoting potential.

## 5 Limitations of the study and future research directions

While this study provides comprehensive insights into the synergistic enhancement of matcha tea with strawberry powder from the Qassim region, several limitations should be acknowledged to guide future investigations.

### 5.1 Limitations

First, the study focused exclusively on the chemical characterization and *in vitro* bioactivities of the extracts, without evaluating their bioavailability or efficacy in biological systems. The antioxidant and anti-inflammatory activities were assessed using cell-free assays (DPPH, ABTS, and protein denaturation inhibition), which, while valuable for preliminary screening, do not fully represent the complex physiological environment where metabolism, absorption, and cellular interactions occur.

Second, the research did not include a sensory evaluation of the formulated beverages. Although the chemical analyses revealed significant enhancements in bioactive compounds and the acidic pH of the strawberry blend (∼3.7) suggests potential flavor modifications, the organoleptic properties, including taste, aroma, color, and overall consumer acceptability, remain unexplored. Sensory characteristics are critical determinants of commercial viability and consumer preference for functional beverages.

Third, the study utilized strawberries from a single growing region (Qassim) and a specific matcha cultivar (Yabukita). While this controlled approach ensured consistency, it limits the generalizability of the findings to other strawberry varieties, matcha cultivars, or geographical origins that may exhibit different phytochemical profiles.

Fourth, the extraction temperatures were limited to three points (5 °C, 70 °C, and 100 °C). Although these represent cold, warm, and boiling conditions, intermediate temperatures (e.g., 40 °C and 85 °C) were not investigated, potentially missing optimal extraction windows for specific compounds.

### 5.2 Future research directions

Future studies should prioritize comprehensive sensory evaluation of the matcha–strawberry blends to assess consumer acceptability. Descriptive sensory analysis should be conducted to characterize flavor profiles, including acidity, bitterness, astringency, and overall palatability across different extraction temperatures. Consumer hedonic testing would provide valuable insights into preferences for cold versus hot preparations, guiding product development for diverse market segments.

*In vivo* studies using animal models and human clinical trials are essential to validate the bioavailability, pharmacokinetics, and therapeutic efficacy of the identified bioactive compounds. Investigating the absorption and metabolism of key synergistically enhanced compounds, particularly catechins and anthocyanins, would elucidate their physiological relevance.

Further research should explore the application of intermediate extraction temperatures (e.g., 40 °C and 85 °C) to identify optimal conditions for balancing the recovery of heat-stable phenolics with the preservation of heat-labile compounds such as vitamin C and anthocyanins. Response surface methodology could be employed to model and optimize multiple bioactive responses simultaneously.

The mechanistic basis of the observed synergistic effects warrants deeper investigation. Molecular studies examining copigmentation interactions, protein-phenolic binding, and pH-mediated stabilization mechanisms would provide fundamental insights into matrix interactions. Spectroscopic techniques such as NMR and FTIR could elucidate the structural basis of compound protection within the strawberry–matcha matrix.

Finally, comparative studies using different strawberry cultivars, matcha grades, and processing methods (e.g., freeze–drying vs. air–drying) would establish the robustness of the synergistic effects and identify optimal ingredient combinations for maximum bioactive enhancement. Shelf-life studies evaluating the stability of formulated beverages under various storage conditions would further support the development of commercial applications.

## Data Availability

The original contributions presented in the study are included in the article/supplementary material, further inquiries can be directed to the corresponding author.
